# The current landscape of adaptive immune receptor genomic and repertoire data: OGRDB and VDJbase

**DOI:** 10.1093/nar/gkaf1094

**Published:** 2025-11-06

**Authors:** William D Lees, Ayelet Peres, Vered Klein, Naama Amos, Uddalok Jana, Eric Engelbrecht, Zachary Vanwinkle, Yaniv Malach, Thomas Konstantinovsky, Pazit Polak, Corey T Watson, Gur Yaari

**Affiliations:** Clareo Biosciences, Louisville, KY 40202, United States; Department of Biochemistry and Molecular Genetics, University of Louisville, Louisville, KY 40202, United States; Department of Pathology, Yale School of Medicine, New Haven, CT 06510, United States; Faculty of Engineering, Bar Ilan University, 5290002 Ramat Gan, Israel; Bar Ilan Institute of Nanotechnology and Advanced Materials, Bar Ilan University, 5290002 Ramat Gan, Israel; Faculty of Engineering, Bar Ilan University, 5290002 Ramat Gan, Israel; Bar Ilan Institute of Nanotechnology and Advanced Materials, Bar Ilan University, 5290002 Ramat Gan, Israel; Faculty of Engineering, Bar Ilan University, 5290002 Ramat Gan, Israel; Bar Ilan Institute of Nanotechnology and Advanced Materials, Bar Ilan University, 5290002 Ramat Gan, Israel; Department of Biochemistry and Molecular Genetics, University of Louisville, Louisville, KY 40202, United States; Department of Biochemistry and Molecular Genetics, University of Louisville, Louisville, KY 40202, United States; Department of Biochemistry and Molecular Genetics, University of Louisville, Louisville, KY 40202, United States; Faculty of Engineering, Bar Ilan University, 5290002 Ramat Gan, Israel; Faculty of Engineering, Bar Ilan University, 5290002 Ramat Gan, Israel; Bar Ilan Institute of Nanotechnology and Advanced Materials, Bar Ilan University, 5290002 Ramat Gan, Israel; Faculty of Engineering, Bar Ilan University, 5290002 Ramat Gan, Israel; Bar Ilan Institute of Nanotechnology and Advanced Materials, Bar Ilan University, 5290002 Ramat Gan, Israel; Department of Biochemistry and Molecular Genetics, University of Louisville, Louisville, KY 40202, United States; Department of Pathology, Yale School of Medicine, New Haven, CT 06510, United States; Faculty of Engineering, Bar Ilan University, 5290002 Ramat Gan, Israel; Bar Ilan Institute of Nanotechnology and Advanced Materials, Bar Ilan University, 5290002 Ramat Gan, Israel

## Abstract

Accurate characterization of adaptive immune receptor repertoires through high-throughput sequencing is critical for understanding immune responses, disease biomarkers, vaccine design, and antibody engineering. However, progress depends on precise knowledge of germline reference sequences, which are challenging to define due to extensive allelic variation and structural complexity in immunoglobulin and T cell receptor loci. To address these challenges, we present major updates to two complementary community resources: the Open Germline Receptor Database (https://ogrdb.airr-community.org) and VDJbase (https://www.vdjbase.org). These resources enable reproducible, evidence-based analysis of immune receptor diversity across populations and species, supporting both fundamental immunology research and precision medicine applications.

## Graphical Abstract

**Graphical Abstract:**
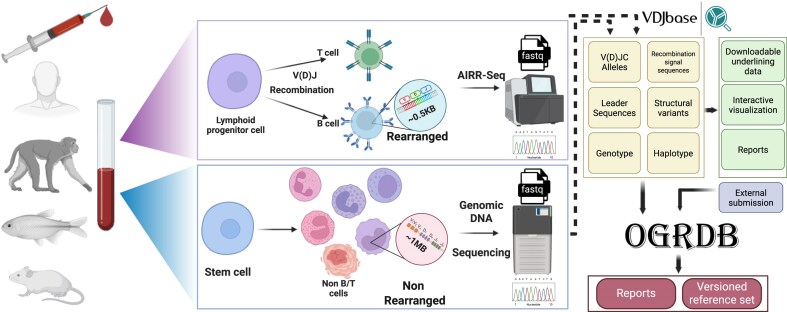

## Introduction

The immune system protects against infection, maintains tissue homeostasis, and enforces tolerance to self [1]. Innate mechanisms provide rapid, pattern-based defense, while the adaptive arm generates highly specific receptors expressed on B and T lymphocytes and retains memory of prior exposure [2, 3]. These receptors are heterodimeric membrane glycoproteins. B cell receptors (BCRs) consist of an immunoglobulin molecule composed of two identical heavy chains paired with two identical light chains [4], whereas T cell receptors (TCRs) are formed by either an $\alpha / \beta$ or a $\gamma / \delta$ heterodimer [5]. Receptor diversity arises during lymphocyte development through a process of recombination with inherent variation, in which variable (V), diversity (D; in heavy, $\beta$ and $\delta$, chains), and joining (J) segments are assembled [6, 7]. In B cells, somatic hypermutation (SHM) and affinity maturation further expand diversity, yielding repertoires on the order of ${\sim} 10^{8}\!-\!10^{10}$ distinct receptor sequences per human individual [8–10].

The study of BCR and TCR repertoires (collectively termed adaptive immune receptor repertoires: AIRRs) spans a wide range of basic scientific questions, including the mechanisms of immune responses [11, 12], clonal dynamics [13, 14], modeling of SHM [15–17], affinity-dependent selection [18–20], and the inference of lineage tree structures [21, 22]. It also has strong translational applications, such as the discovery of biomarkers for disease [23–27], rational vaccine design [28, 29], and antibody engineering [30, 31]. Progress in this field relies on high-resolution characterization of AIRRs, which can be generated using diverse experimental sequencing platforms (AIRR-seq). These range from bulk DNA and RNA sequencing approaches of AIRR-seq that provide scale to single-cell assays that recover paired chains and connect receptors to phenotype [32].

Biological insights from AIRR-seq rely on accurate characterization of the inherited germline components. The loci encoding BCRs and TCRs are complex [33], with many allelic variations [34–36], frequent copy number and structural variation [37, 38], and pseudogenes [39]. Incomplete or inaccurate references bias gene assignment [40], clonotype definition, and mutation calling, with the greatest impact in underrepresented populations and in species with sparse references [41, 42]. Short-read whole genome approaches often struggle in these regions [43], whereas targeted long-read sequencing and careful quality control improve coverage of non-rearranged sequences and reduce annotation error [39].

Across both genomic and repertoire data, open and versioned workflows are essential [44–46]. Community guidelines, standardized formats, and reproducibility enable meaningful cross-study comparisons and meta analyses [47]. To facilitate transparent, evidence-based community resources that supply curated information about germline variations in experimental datasets, the Open Germline Receptor Database (OGRDB) [48] and VDJbase [49] were developed. OGRDB provides rigorous curation of individual germline sequences with community review, while VDJbase organizes population-level genotypes, haplotypes, and usage patterns from curated experimental datasets with interactive visualizations and reports (Graphical abstract). Since their initial publications, both have incorporated genomic long-read sequences, flanking regions, and matched genomic–repertoire datasets, creating opportunities to probe variation across populations and species and to support both basic research and precision applications.

## VDJbase

**Figure 1.**
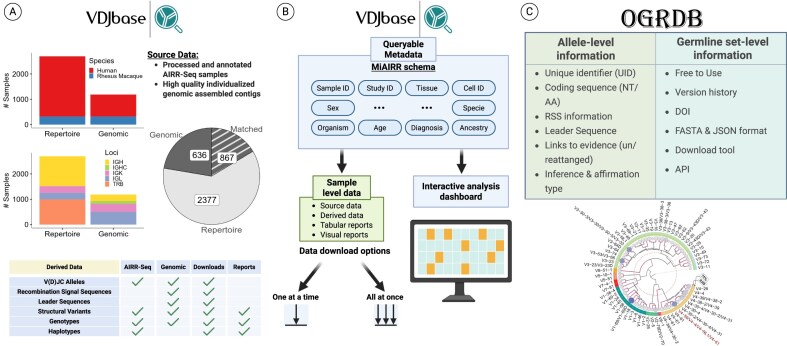
Data organization and accessibility in VDJbase and OGRDB. (**A, B**) Source data include processed and annotated AIRR-seq repertoires as well as high-quality individualized genomic assemblies. From these, derived data products are generated following the MiAIRR schema, encompassing source and derived tables, tabular and visual reports, and interactive dashboards. Sample-level metadata (e.g. study, tissue, diagnosis, and ancestry) are fully queryable, with flexible options for data exploration and download. (**C**) Allele-level and germline set-level information are provided, including coding sequences, recombination signal sequences (RSSs), leader sequences, evidence links, and inference/affirmation details, all available under a CC0 license in AIRR-compliant formats, spanning multiple species and loci. Created in BioRender. Peres, A. (2025) https://BioRender.com/uwdm9vl.

VDJbase contains a large collection of queryable biological information mined from carefully curated AIRR-seq and genomic long-read sequencing data. Samples in VDJbase are either bulk-sequenced AIRR-seq repertoires or long-read genomic assemblies of IG/TR loci. To date, it holds >3000 human and 600 rhesus macaque samples (Fig. 1A), which span all human immunoglobulin loci, with entries for IGH ($n=1436$), IGK ($n=580$), IGL ($n=766$), IGHC ($n=105$), and TRB ($n=993$) (Fig. 1A). Of these, 636 are genomic samples and 2377 are AIRR-seq samples. The collection also provides matched genomic and repertoire datasets of rhesus macaque across IGH, IGK, and IGL (${>}200$ samples at each locus), along with a parallel human cohort of ${>}170$ matched individuals. This represents a substantial expansion from the initial release of VDJbase, which contained just over 500 human AIRR-seq samples from 15 studies and no genomic or nonhuman primate data [49]. For human genomic entries, super population labels are documented, and for rhesus macaque, colony origins are documented. This enables unique data stratification of allele and genotype summaries by broad ancestry groups.

From each AIRR-seq sample, we infer the set of V(D)JC germline alleles (the genotype), reconstruct a haplotype when sufficient information is available [50], and identify genes that are absent from the sample [37]. From genomic contigs assembled from long-read sequencing data, we extract the genotype [51] and flanking leader and RSSs. Structural features and quality metrics are recorded where available. These outputs are accessible as tables, visual reports, and through an enhanced interface with interactive visualizations and a dedicated genome browser (Fig. 1B), allowing rapid, intuitive exploration of allele usage, abundance, genotype comparisons, and haplotype structures.

The MiAIRR standard defines essential metadata for AIRR-seq studies [52]. A MiAIRR-compliant, queryable metadata system enhances analysis and discovery, enabling filtering by species, locus, chain, data type (repertoire or genomic), study, tissue, diagnosis, ancestry, and super population, with export of filtered result sets for downstream analysis (Fig. 1B). All underlying source data, processed and curated with a FAIR (findable, accessible, interoperable, reusable)-compliant Nextflow pipeline in VDJbase (repertoires and genomic contigs), are available for download individually or via the Application Programming Interface (API) at https://madc.vdjbase.org/. Downloads and API access are open, with content released under a CC0 license and no login required.

## OGRDB

OGRDB was initially developed as a curation tool for identifying and publishing newly discovered receptor germline sequences [48] but has been extended to encompass the management and publication of community-curated germline sets [53]. Work is conducted in close coordination with the AIRR Community and with the T cell Receptor and Immunoglobulin Nomenclature Sub Committee of the International Union of Immunological Societies (IUIS). OGRDB accepts submissions of germline sets that meet or enhance current evidential standards for a specific species, and sequences are submitted to IUIS for ratification where IUIS evidential standards are met [54]. To date, OGRDB hosts published germline sets for the human IGH, IGK, and IGL loci and for 17 laboratory mouse strains. A complete human TCR set covering TRA, TRB, TRG, and TRD is in preparation, as are sets for several additional species. In this way, OGRDB has moved beyond its original focus on individual inferred alleles to provide complete, community-curated germline sets across species, representing a major expansion in both scale and scope.

Each allele record includes detailed provenance with links to public sequence repositories, so that users can inspect the supporting evidence for every sequence and view linked records in VDJbase. Reference sets are fully versioned, with change records maintained between releases. All versions are archived at Zenodo for long term storage, and a DOI is issued for each release.

To promote use, sets are distributed in multiple formats with guidance and command line utilities for common aligners and pipelines. An OpenAPI 3 interface enables programmatic exploration and download, which facilitates integration with custom analyses and automated updates.

Access remains fully open. Published content is available without login and under a CC0 license. Allele entries are backed by records in repositories such as GenBank and the Sequence Read Archive. The platform supports multiple curation teams working across loci and species with minimal centralized overhead, and the source code is available under an open source license.

## Use cases

**Figure 2.**
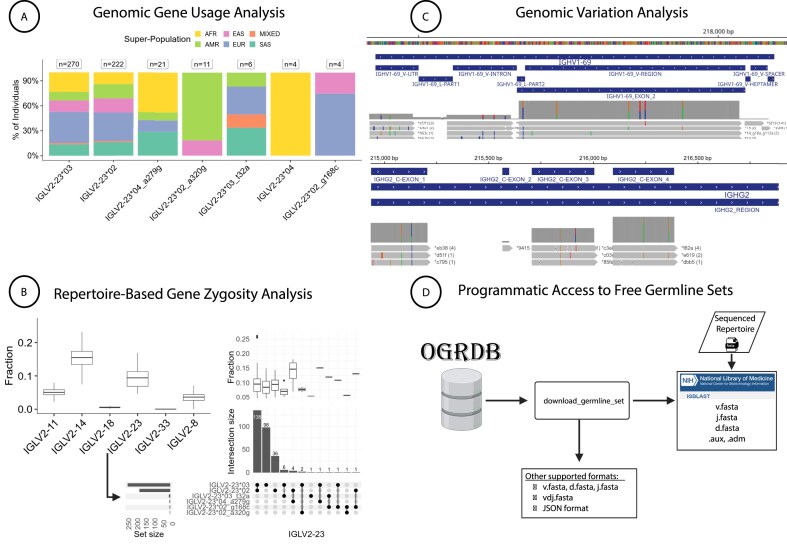
**A**) Genomic gene usage analysis across super populations, showing the distribution of alleles in individuals found in VDJbase [55]. (**B**) Repertoire-based gene zygosity analysis highlights allele presence and absence patterns across expressed repertoires, illustrating shared and unique zygosity profiles from annotated IGL repertoires in VDJbase. (**C**) Genomic data captured in VDJbase reveal extensive variation in both coding and regulatory regions, which can be examined in the browser. Here are shown examples from the human IGHV (upper rows) and IGHC (lower rows) loci [38, 56]. (**D**) A command-line utility allows germline sets to be downloaded in the exact format required by NCBI’s IgBLAST tool [57], making it easy to construct pipelines and to keep them up to date with reference set revisions. The utility also provides information in a flexible range of other formats, for use with other tools and in custom analysis. Created in BioRender. Peres, A. (2025) https://BioRender.com/gbjevp8

We demonstrate the utility of the VDJbase and OGRDB platforms through several representative use cases, highlighting how curated genomic and AIRR-seq datasets can drive discovery and support diverse analytical workflows (Fig. 2).

A first use case is population-level gene usage, which in humans can be explored across super populations using curated genomic datasets in VDJbase. Stratification by super population reveals population-specific alleles and alleles that are common across super populations (Fig. 2A). For example, analysis of IGLV2-23 shows that allele *01, the first to be described by IMGT, is absent in the entire cohort. Moreover, the newly discovered IGLV2-23*02_a320g appears only in individuals from two super populations (AMR and EAS), and another newly discovered allele (IGLV2-23*04_a279g) is more prevalent in AFR and SAS. This functionality enables rapid assessment of germline diversity across global cohorts using intuitive visualizations and accessible underlying data.

A second use case is repertoire-based zygosity analysis, which provides a complementary view of expressed gene variation (Fig. 2B). Using the same gene, IGLV2-23, repertoire data can be used to calculate allele usage frequencies and zygosity profiles. An UpSet plot of IGL repertoires reveals combinations of alleles observed in individuals, including the presence and absence patterns across samples. For individuals with matched genomic and AIRR-seq data, repertoire-inferred zygosity can be compared directly to the underlying germline genotype. This integrated approach supports cross-validation and highlights potential discrepancies between genotype and expression.

A third use case is fine-scale exploration of genomic variation through the browser-based viewer (Fig. 2C). This interface allows inspection of both coding and regulatory elements, including known and novel variants. IGHV and IGHC loci exhibit extensive polymorphism, including variation supported by long-read sequencing across large cohorts [38, 56].

A fourth use case is reference set retrieval, supported by a dedicated command-line utility that outputs germline sequences in multiple formats, including IgBLAST-compatible segment FASTAs, combined VDJ sets, and JSON-formatted outputs (Fig. 2D). These files can be used directly in annotation pipelines and are kept synchronized with curated, versioned reference sets. The utility also supports downloading auxiliary metadata, and OGRDB provides programmatic access through public APIs. This facilitates integration into automated workflows and enables scalable, reproducible immunogenetics analysis.

## Future directions

Looking ahead, we see several important directions for further development and community involvement. First, we encourage the research community to contribute datasets and to join ongoing efforts to expand the available resources. Broad participation will help ensure that germline reference sets and repertoire datasets reflect the full diversity of human and nonhuman populations.

Second, we will continue curating both AIRR-seq and genomic datasets, with an emphasis on generating high-quality, carefully validated resources. This work extends beyond human data to include other species of biomedical importance, where curated germline references remain sparse.

Third, we are collaborating with the IUIS Nomenclature Committee to refine standards for allele submission and naming conventions. This collaboration supports the continued production of high-confidence, community-approved reference sets in OGRDB and their integration with widely used analysis tools.

Finally, we aim to enhance interoperability and accessibility by strengthening APIs, building additional visualization and analysis tools, and integrating with broader FAIR data initiatives. These efforts will make it easier to connect germline references and repertoires with downstream analysis pipelines, enabling reproducible and large-scale immunogenetics research. Both databases are integrated into the AIR Knowledge Commons [58], an emerging network of AIR-related resources, which enables unified access to multiple sources of AIR data, including repertoires, antigen specificity, and germline genes and loci.

## Data Availability

The OGRDB is available at https://ogrdb.airr-community.org. The VDJbase is available at https://www.vdjbase.org.
